# Effects of Genistein and Exercise Training on Brain Damage Induced by a High-Fat High-Sucrose Diet in Female C57BL/6 Mice

**DOI:** 10.1155/2022/1560435

**Published:** 2022-05-17

**Authors:** Rongzi Li, Xiaowen Ding, Thangiah Geetha, Moni Fadamiro, Chaheyla R. St Aubin, Minsub Shim, Layla Al-Nakkash, Tom L. Broderick, Jeganathan Ramesh Babu

**Affiliations:** ^1^Department of Nutrition, Dietetics, And Hospitality Management, Auburn University, AL 36849, USA; ^2^Department of Physiology, College of Graduate Studies, Midwestern University, AZ 85308, USA; ^3^Department of Biochemistry, College of Graduate Studies, Midwestern University, AZ 85308, USA; ^4^Department of Physiology, Laboratory of Diabetes and Exercise Metabolism, College of Graduate Studies, Midwestern University, AZ 85308, USA

## Abstract

In recent decades, a shift in the nutritional landscape to the Western-style diet has led to an unprecedented rise in the prevalence of obesity and neurodegenerative diseases. Consumption of a healthy diet and engaging in regular physical activity represents safe and affordable approaches known to mitigate the adverse consequences of the Western diet. We examined whether genistein treatment, exercise training, and a combination treatment (genistein and exercise training) mitigated the effects of a Western diet-induced by high-fat, high-sugar (HFHS) in brain of female mice. HFHS increased the amyloid-beta (A*β*) load and phosphorylation of tau, apoptosis, and decreased brain-derived neurotrophic factor (BDNF) levels. Exercise training and genistein each afforded modest protection on A*β* accumulation and apoptosis, and both increased BDNF. The greatest neuroprotective effect occurred with combination treatment. BDNF and all markers of A*β* accumulation, phosphorylation of tau, and apoptosis were improved with combined treatment. In a separate series of experiments, PC12 cells were exposed to high glucose (HG) and palmitate (PA) to determine cell viability with genistein as well as in the presence of tamoxifen, an estrogen receptor antagonist, to assess a mechanism of action of genistein on cell apoptosis. Genistein prevented the neurotoxic effects of HG and PA in PC12 cells and tamoxifen blocked the beneficial effects of genistein on apoptosis. Our results indicate the beneficial effects of genistein and exercise training on HFHS-induced brain damage. The benefits of genistein may occur via estrogen receptor-mediated pathways.

## 1. Introduction

Over the past decades, advances in food technology introduced increased affordability and availability of fast foods in the form of refined grains and sugars [1]. As a result, the dietary composition has been shifted from diets high in fiber and complex carbohydrates to energy-rich diets consisting of simple sugars, refined grains, and high-fat foods: A dietary pattern known as the “Western diet” [2]. The Western diet has been associated with adverse effects on general health, including the development of insulin resistance, and an increased prevalence of obesity, type 2 diabetes mellitus (T2DM), and cardiovascular diseases (CVD) [3, 4]. Moreover, emerging evidence has documented the damaging effects of the Western diet on the brain [5, 6]. Both human epidemiological and animal studies have shown that chronic intake of a high-fat, high-sugar (HFHS) diet is associated with declined cognitive function, impaired performance on cognitive tests, and increased risk of neurodegenerative diseases [5–7].

Increasing the intake of bioactive compounds in habitual diets has been suggested as a potential dietetic treatment to improve brain health. Among the different bioactive compounds, genistein has exhibited the ability to protect the brain from oxidative stress and improve synapse development and cognitive function [8, 9]. Genistein belongs to the class of soy isoflavones and is found in soy-based foods. Evidence from animal and human studies supports the notion that chronic genistein consumption may offer protection against a broad range of human conditions, including menopausal symptoms, T2DM, CVD, and neurodegenerative conditions such as Alzheimer's disease (AD) [10–12]. Genistein possesses a structure that is similar to estrogen and therefore can bind the estrogen receptor (ER) [12]. However, unlike the traditional hormone replacement therapy, which increases the risk of breast and ovarian cancers, genistein is an alternative medicine that is not associated with a higher risk of cancers [13, 14]. In addition, genistein is an attractive option because reduced circulating levels of estrogen are associated with high levels of A*β* levels observed in female patients with AD, indicating a role in the pathobiology of AD [15–17]. Another important neuroprotective role of this isoflavone is supported by the observation that genistein exerts anti-apoptotic actions via ER-mediated mechanisms [18, 19].

Exercise is a safe nonpharmacologic and affordable alternative for the treatment of different pathological conditions and maintenance of general health. Exercise training has been shown to improve memory and cognitive processes and prevent the development of toxic species of amyloid-beta (A*β*) in the brain of the 3xTg mouse model of AD [20]. Given that both genistein and exercise training offer protection against the development of neurodegenerative diseases, it remains unclear whether supplemental genistein or exercise training affords protection against HFHS feeding-induced brain damage in the mouse. Whether combined treatment with genistein and exercise offers additional protection compared to either treatment alone against HFHS feeding also remains to be determined.

In this study, the effects of 12 weeks of genistein treatment, exercise training, and combined treatment on markers of A*β* deposition, phosphorylation of tau, neurogenesis, and apoptosis were determined in brain from HFHS-fed female mice. Secondly, to determine a possible mechanism of action of genistein, PC12 cells were exposed to palmitic acid (PA, 16 : 0) and high glucose (HG) concentrations in the presence of genistein and tamoxifen. These conditions were chosen to determine if the protective effect of genistein against HG and PA-induced neural toxicity involves an ER-mediated downregulation of apoptotic signaling.

## 2. Materials and Methods

### 2.1. Mouse Model, Diet and Exercise Training

Fifty C57BL/6 female mice (Charles River Laboratories, Wilmington, MA, USA) aged 6 weeks were used in the study. Female mice were selected because the prevalence of neurological diseases, including AD, is higher in the female population compared to the male population. In addition, the female population exhibits a greater deterioration in cognitive function compared to age-matched males [21]. Mice were assigned to five groups (*n* = 10/group): (1) control, (2) HFHS diet, (3) HFHS diet + exercise, (4) HFHS diet + genistein, and (5) HFHS diet + exercise + genistein. The HFHS diet consisted of 60% fat (32.3 g/kg of corn oil and 316.6 g/kg of lard), 20% protein, 20% carbohydrate (Dyets Inc. Bethlehem, PA, USA), and 42 g/L of sugar dissolved in the drinking water (55% fructose/45% sucrose). This diet is known to induce significant insulin resistance and visceral obesity in the C57BL/6 mouse [22]. Control mice were fed with a standard diet that contained 5 g fat, 20.3 g protein, 66 g carbohydrate, and regular drinking water. Exercise training consisted of moderate-intensity treadmill running for 30 min/day, 5 days/week. Genistein treatment was incorporated into the food pellet at 600 mg genistein/kg (Dyets Inc., PA, USA). This genistein dose is sufficient to produce significant increases in plasma levels of free genistein [23]. Mice were maintained in a room with a 12-hour light/dark period and at a temperature of 22 °C. After 12 weeks of treatment, mice were sacrificed, and brain tissue was frozen in liquid nitrogen for western blotting analyses. The animal protocols described in this study were approved by the Institutional Animal Care and Use Committee at Midwestern University. Guidelines in the National Institutes of Health's Guide for the Care and Use of Laboratory Animals were followed.

### 2.2. Cell Culture and Treatment

Undifferentiated PC12 cells (ATCC® CRL-1721™) were maintained in RPMI-1640 medium containing 10% heat-inactivated horse serum, 5% fetal bovine serum (FBS), and penicillin/streptomycin (50 units/50 *μ*g of each per mL) at 37 °C with 95% air–5% CO_2_. The culture medium was replaced every 2–3 days. PC12 cells were differentiated by exposure to 50 ng/mL mouse nerve growth factor (Alomone Laboratories, Israel) for 5-7 days in RPMI-1640 medium supplemented with 1% FBS and penicillin/streptomycin (described hereafter as differentiation medium).

To assess the effects of glucose and fatty acids on cell viability, PC12 cells were exposed to either 13.5 mg/ml glucose (HG) or 0.3 mM palmitic acid (PA) bound to 0.15 mM fatty acid-free bovine serum albumin (BSA) or 13.5 mg/ml glucose in the presence of PA. In a separate experiment, PC12 cells were pretreated with genistein (2, 5, 10, 50, and 100 *μ*M) alone or in combination with the estrogen receptor antagonist tamoxifen (5 *μ*M) for 2 h in differentiation medium, followed by HG and PA treatment to determine whether the cytotoxic effects can be prevented.

### 2.3. Preparation of BSA-Bound Fatty Acids

PA (Santa Cruz 215881) was conjugated to fatty acid-free BSA (Sigma A8806). Following previously described methods [24], PA was dissolved in 100% ethanol at 70 °C to make a 0.3 mM stock solution. The PA-BSA was dissolved in a differentiation medium to make a 0.15 mM solution and then sterilized by filtering through a 0.2 mM filter. The molar ratio of PA and BSA for the experiments was 2 : 1. For control, PC12 cells were treated with a differentiation medium containing 150 *μ*M PA-free BSA and 0.1% ethanol.

### 2.4. Determination of Cell Proliferation

The proliferation of PC12 cells was assessed by MTT assay. PC12 cells were seeded in 96-well plates at the initial density of 3000 cells per well and then differentiated for 5-7 days before the treatment. Twenty-four hours after treatment, the medium was discarded, and 50 *μ*L of serum-free medium and 50 *μ*L of MTT Reagent were added into each well, followed by incubation for 3 h. Then, the supernatant was discarded and 150 *μ*L of MTT Solvent was added into each well. A microplate reader determined the absorbance at 590 nm. Cell proliferation rate was calculated from the absorbance value.

### 2.5. Western Blot Analysis

Triton lysis buffer (50 mM Tris-HCl [pH 7.5], 150 mM NaCl, 10 mM NaF, 0.5% Triton X-100, 1 mM Na_3_VO_4_, 1 mM phenylmethylsulfonyl fluoride, and 2 *μ*g/mL leupeptin, aprotinin, and protease inhibitor cocktail) was used to homogenize whole brain samples. The cells were harvested in lysis buffer containing 20 mM Tris-HCl (pH 7.5), 150 mM NaCl, 1 mM Na_2_EDTA, 1 mM EGTA, 1% Triton, 2.5 mM sodium pyrophosphate, 1 mM beta-glycerophosphate, 1 mM Na_3_VO_4_, and 1 *μ*g/ml leupeptin. Protein concentrations of either brain homogenate or cell lysates were measured by the Pierce 660-nm protein assay reagent (Thermo Scientific, Rockford, IL). The samples were run on 8–15% sodium dodecyl sulfate-polyacrylamide gel electrophoresis (SDS–PAGE) gels. The proteins were transferred onto polyvinylidene difluoride membranes. Transferred membranes were blocked for one hour in Tris-buffered saline Tween-20 (TBST) containing 5% milk. The blots were then incubated with various primary antibodies in TBST at 4 °C overnight. After washing, the blots were incubated with secondary antibodies for two hours at room temperature. The enhanced chemiluminescence reagents were used to visualize the immunoreactive bands.

### 2.6. Statistical Analysis

Quantification of Western blot data was performed by Image J software and data were analyzed using GraphPad Prism. A one-way analysis of variance followed by Tukey's test was used to determine differences in group means. All values are reported as mean ± SEM. A *p* < 0.05 was considered statistically significant.

## 3. Results

The metabolic data from mice fed an HFHS diet were previously published [25]. Briefly, mice fed an HFHS diet for 12 weeks had significantly greater body mass, higher plasma glucose, insulin, and IL-6 levels than control mice fed a standard diet (*p* < 0.05). HFHS diet-fed mice treated with genistein, either alone or combined with exercise, had significantly decreased body mass and plasma glucose levels than HFHS controls.

### 3.1. Genistein or Genistein with Exercise Reduces the Accumulation of A*β* and Hyperphosphorylated Tau in HFHS Diet-Fed Mice

**Figure 1:**
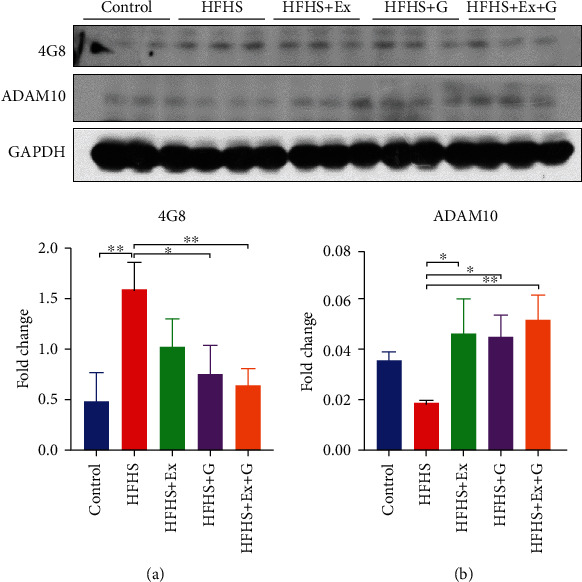
Effect of genistein, exercise training, and combination treatment on A*β* deposition in female mouse brain. (a) Representative blot images with the corresponding densitometry measurement of 4G8. (b) Representative blot images with the corresponding densitometry measurement of ADAM10. HFHS: high-fat, high-sugar diet; HFHS + Ex: HFHS + exercise; HFHS + G: HFHS + genistein; HFHS+Ex+G: HFHS + exercise + genistein. Data are presented as mean ± SEM for 3 mice per group. ∗*p* ≤ 0.05 and ∗∗*p* ≤ 0.01.

**Figure 2:**
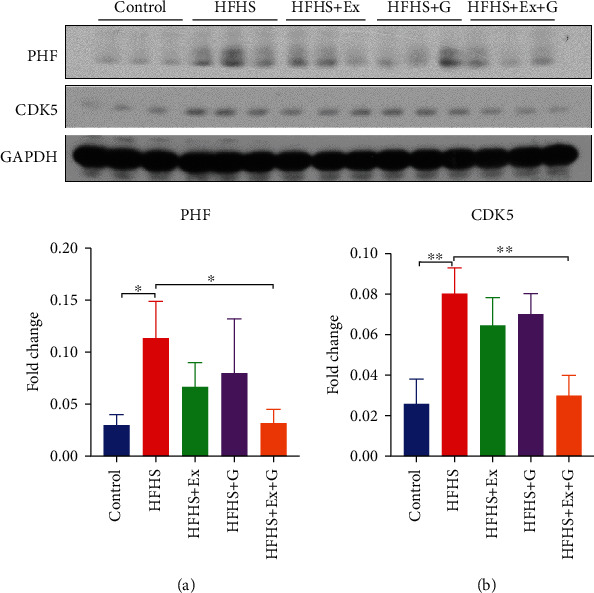
Effect of genistein, exercise training, and combination treatment on phosphorylation of tau in female mouse brain. (a) Representative blot images with the corresponding densitometry measurement of PHF. (b) Representative blot images with the corresponding densitometry measurement of CDK5. HFHS: high-fat, high-sugar diet; HFHS + Ex: HFHS + exercise; HFHS + G: HFHS + genistein; HFHS+Ex+G: HFHS + exercise + genistein. Data are presented as mean ± SEM for 3 mice per group. ∗*p* ≤ 0.05 and ∗∗*p* ≤ 0.01.

Evidence suggests that increased intake of saturated fat and sugar in the diet may increase the risk of AD [26]. Amyloid plaques and neurofibrillary tangles (NFTs) are two main pathological conditions of AD. Amyloid plaques are the extracellular accumulation of the A*β*, and the NFTs are the intracellular deposits of the hyperphosphorylated tau protein. To examine how genistein affects HFHS diet-induced changes in AD neuropathology, we measured the levels of A*β* and hyperphosphorylated tau in the different treatment groups. Protein expression levels of A*β* detected by 4G8 and expression levels of hyperphosphorylated tau detected by PHF antibody are illustrated in Figures 1 and 2, respectively. As expected, the HFHS diet significantly increased the level of A*β* protein and hyperphosphorylated tau protein. The treatment with genistein or genistein with exercise was associated with a reduction of A*β* compared to HFHS-fed mice. Although genistein or exercise alone did not significantly change the level of hyperphosphorylated tau, the combination treatment was effective in reducing both 4G8 and PHF levels compared to brains from the HFHS diet treatment group.

A disintegrin and metalloprotease 10 (ADAM10) is an *α*-secretase responsible for the non-amyloidogenic pathway of amyloid precursor protein (APP). We observed a decreased expression of ADAM10 in brain tissue of mice fed HFHS compared to the control mice while treating with genistein and/or exercise improved levels of ADAM10 (Figure 1). Cyclin-dependent kinase 5 (CDK5) is an essential kinase involved in the phosphorylation of tau protein. We found a significant increase in the level of CDK5 in the HFHS group, while combining exercise and genistein treatment returned the CDK5 level to that of control mice.

### 3.2. Genistein and/or Exercise Increases the Expression of BDNF in HFHS Diet-Fed Mice

**Figure 3:**
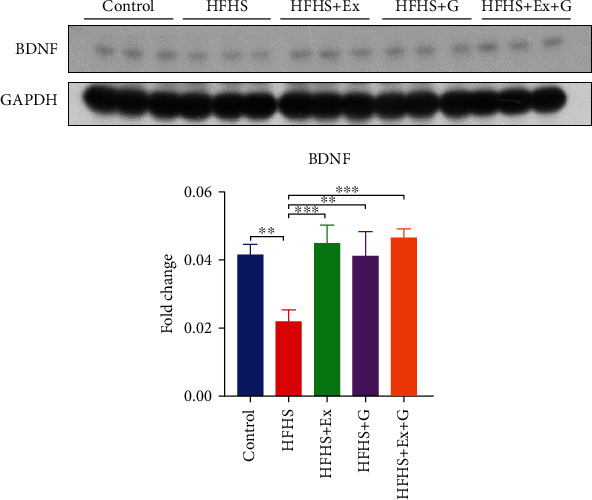
Effect of genistein, exercise training, and combination treatment on BDNF expression in female mouse brain. HFHS: high-fat, high-sugar diet; HFHS + Ex: HFHS + exercise; HFHS + G: HFHS + genistein; HFHS+Ex+G: HFHS + exercise + genistein. Data are presented as mean ± SEM for 3 mice per group. ∗∗*p* ≤ 0.01 and ∗∗∗*p* ≤ 0.001.

Brain-derived neurotrophic factor (BDNF), a key molecule involved in synapse function and neurite growth, is emerging as a crucial player in synaptic plasticity, learning, and memory [27]. Our results show that the protein level of BDNF in the brain of mice fed an HFHS diet was reduced compared to mice fed a control diet (Figure 3). Treatment with genistein alone or exercise alone resulted in a significant increase in the BDNF level compared to HFHS diet-treated mice. Combination treatment similarly improved the expression of BDNF.

### 3.3. Genistein or Genistein with Exercise Decreases Apoptosis in HFHS Diet-Fed Mice

**Figure 4:**
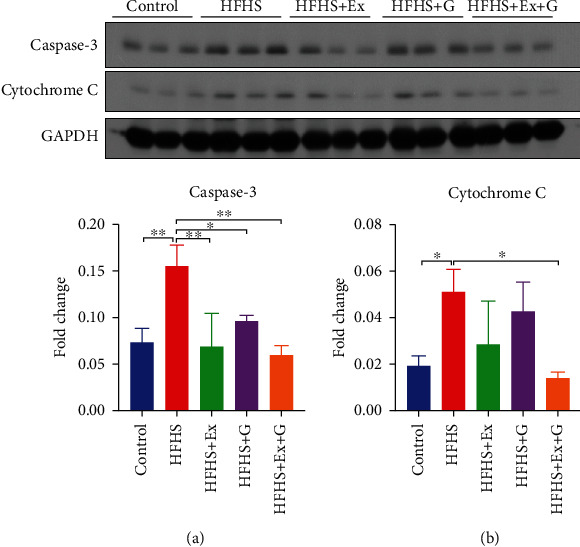
Effect of genistein, exercise training, and combination treatment on apoptosis in female mouse brain. (a) Representative blot images with the corresponding densitometry measurement of caspase-3. (b) Representative blot images with the corresponding densitometry measurement of cytochrome C. HFHS: high-fat, high-sugar diet; HFHS + Ex: HFHS + exercise; HFHS + G: HFHS + genistein; HFHS+Ex+G: HFHS + exercise + genistein. Data are presented as mean ± SEM for 3 mice per group. ∗*p* ≤ 0.05 and ∗∗*p* ≤ 0.01.

Moreover, we evaluated the effects of genistein and exercise training on the apoptotic process in HFHS-fed mice. The two main pathways of apoptosis are intrinsic (mitochondria) and extrinsic (death ligand) pathways. Cytochrome c plays a vital role in the intrinsic pathway and can trigger the activation cascade of caspases [28]. Our results indicate that the HFHS diet led to an increase in cytochrome C levels, while genistein and exercise combination treatment returned cytochrome C levels to control levels. Caspase-3 is an executioner caspase in apoptosis due to its role in regulating the destruction of cellular structures or the degradation of cytoskeletal proteins [29]. We found that the expression of activated caspase-3 was significantly increased in the brain of mice fed an HFHS diet (Figure 4). However, the addition of genistein or exercise or the combination treatment reversed caspase-3 levels to normal.

### 3.4. Incubation of PC12 Cells with HG and PA Results in Loss of Viability

**Figure 5:**
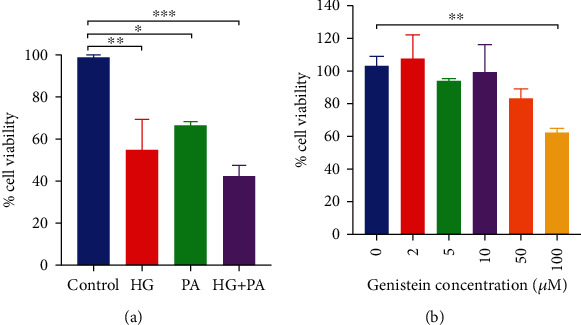
(a) Effect of high glucose, palmitate and combined high glucose and palmitate on PC12 cell viability. (b) Effect of genistein on PC12 cell viability. HG: high glucose; PA: palmitate; HG + PA: high glucose + palmitate. Data are presented as mean ± SEM. ∗*p* ≤ 0.05, ∗∗*p* ≤ 0.01, and ∗∗∗*p* ≤ 0.001.

Differentiated PC12 cells were treated with HG or PA or co-treated with HG and PA for 24 hours. The effect of HG and PA on PC12 cell viability was then determined using an MTT assay. As shown in Figure 5(a), PC12 cell viability was significantly decreased by the presence of HG and PA. The combined treatment of HG and PA, which significantly decreased cell viability to 40% (*p* < 0.01), was selected for subsequent experiments. The effects of different concentrations of genistein on cell viability were also investigated. Genistein induced no cytotoxic effects at concentrations of 2, 5, 10, and 50 *μ*M (Figure 5(b)). At the highest concentration used, genistein significantly decreased cell viability compared to control conditions (*p* < 0.05).

### 3.5. Genistein Attenuates HG and PA-Induced Loss of Cell Viability

**Figure 6:**
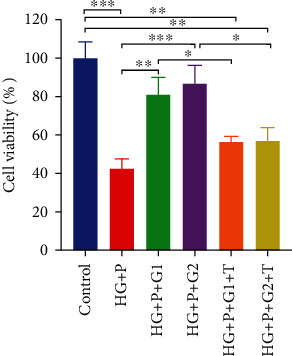
Effect of genistein alone or with tamoxifen on cell viability in high glucose and palmitate-treated PC12 cells. HG + P: high glucose + palmitate; HG + P + G1: high glucose + palmitate +2 *μ*M genistein; HG + P + G2: high glucose + palmitate + 5 *μ*M genistein; HG + P + G1 + T: high glucose + palmitate + 2 *μ*M genistein + tamoxifen; HG + P + G2 + T: high glucose + palmitate + 5 *μ*M genistein + tamoxifen. Data are presented as mean ± SEM. ∗*p* ≤ 0.05, ∗∗*p* ≤ 0.01, and ∗∗∗*p* ≤ 0.001.

In a separate experimental group to examine the effect of genistein on HG and PA-induced cytotoxicity, PC12 cells were pre-treated with 2 *μ*M or 5 *μ*M genistein and then incubated in the HG and PA medium. The viability of PC12 cells pretreated with genistein improved significantly compared with cells that underwent treatment with HG and PA alone (*p* < 0.05). We further examined cell viability when the cells were pretreated with genistein and tamoxifen (ER antagonist) to determine whether the genistein-induced proliferative effect in HG and PA-containing medium was associated with the activation of the ER. As shown in Figure 6, the cell viability of the group pretreated with genistein and tamoxifen was significantly lower than the group pretreated with genistein alone. These results indicated that genistein may exert its proliferative effect in HG and PA-containing medium, partially through the ER-mediated pathway.

### 3.6. Genistein Protects against HG and PA-Induced Apoptosis in PC12 Cells via an ER-Mediated Mechanism

**Figure 7:**
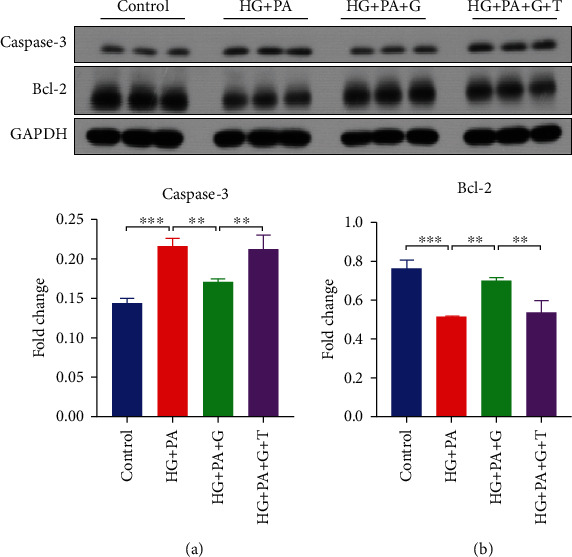
Effect of genistein alone or with tamoxifen on high glucose and palmitate-induced apoptosis in PC12 cells. (a) Representative blot images with the corresponding densitometry measurement of caspase-3. (b) Representative blot images with the corresponding densitometry measurement of Bcl-2. HG + PA: high glucose + palmitate; HG + PA + G: high glucose + palmitate + 5 mM genistein; HG + PA + G + T: high glucose + palmitate + 5 mM genistein + tamoxifen. Data are presented as mean ± SEM for 3 mice per group. ∗∗*p* ≤ 0.01 and ∗∗∗*p* ≤ 0.001.

Control, HG + PA, HG + PA + genistein (HG + PA + G), and HG + PA + genistein + tamoxifen (HG + PA + G + T) were harvested into lysis buffer for western blotting analysis. The expression of caspase-3 and Bcl-2 was determined.  Figure 7 shows significant caspase-3 activation following 24 hours of HG and PA exposure (*p* < 0.01). Preincubation with 5 *μ*M of genistein prevented the increase in caspase-3 activation. The level of activated caspase-3 in genistein pretreated groups was not significantly different from that of the control group. Moreover, as shown in Figure 7(a), the protein levels of caspase-3 in the HG + PA + G + T group were significantly higher than HG + PA + G–treated cultures (*p* < 0.05) and were similar to that in HG + PA-only treated cultures. Bcl-2 western blotting results presented in Figure 7(b) demonstrate that genistein added to the cell culture medium significantly (*p* < 0.05) improved the downregulation of Bcl-2 expression, an effect shown to occur within the HG + PA exposure in PC12 cell cultures. However, the addition of tamoxifen significantly attenuated genistein effects on Bcl-2 expression. These results indicate that genistein inhibits the HG + PA-induced caspase-3 activation and Bcl-2 downregulation via an ER-mediated mechanism, as the addition of tamoxifen returned caspase-3 and Bcl-2 levels to that of HG + PA-only treated cultures.

## 4. Discussion

Consumption of western-style diets high in saturated fat and simple carbohydrates is not only linked to the development of obesity and T2DM but also exacerbates these metabolic conditions. These obesity-generating diets also increase the risk of the early development of age-related brain disorders as well as the risk for cognitive dysfunction and decline, dementia, and AD [30]. Lifestyle interventions in the form of regular physical activity are a safe and widely modifiable factor known to improve health outcomes. Several large-scale clinical studies have demonstrated the benefits of regular physical activity on metabolic and cardiovascular health. In the recent decade, there has been an increased focus on the benefits of exercise as an important treatment for patients with neurodegenerative diseases [31]. Since the consumption of diets high in fats and sugars is also considered a preventable modifiable risk factor, other effective lifestyle alternatives exist for the improvement of brain health. For instance, consumption of plant-based soy-rich foods, including the isoflavone genistein, has been associated with improved cognitive function, cerebral blood flow, and metabolism [32–36]. Experimentally, genistein has been extensively studied in male and female mice as a promising therapy for the treatment of obesity and diabetes-induced cognitive dysfunction with similar efficacy because of its reported anti-inflammatory, anti-apoptotic, and antioxidant properties [32]. In addition, we have recently demonstrated a robust neuroprotective effect of genistein in a model of severe obesity [37]. In fact, four weeks of genistein treatment improved insulin signaling, increased expression of BDNF, and decreased the A*β* load and hyperphosphorylated tau in the brain of ob/ob mice [37]. In the present study, the effects of exercise training and genistein supplementation on HFHS-induced brain injury were determined. Because the prevalence of AD is higher in women compared to age-matched men [21], we chose to use female mice for this study. The neuroprotective effects of genistein on the male brain are of major interest and have been reported [38–44].

We found that chronic consumption of an HFHS diet increased the level of A*β* protein and tau phosphorylation in the brain tissue of mice. These are well-documented hallmarks of AD and are consistent with previous studies [26, 45]. The HFHS diet used in this study was sufficient to induce visceral obesity, hyperglycemia, and insulin resistance in the C57BL/6 mice [25]. Increasing evidence suggests that insulin resistance is associated with AD pathology. It is proposed that insulin could regulate the levels of A*β* by modulating the balance between A*β* production and degradation. Also, defect in the insulin signaling pathway, which is a feature observed with insulin resistance and obesity [22], may promote the phosphorylation of tau protein through activation of glycogen synthase kinase 3 [46]. In addition, impaired insulin signaling leads to mitochondrial dysfunction, resulting in increased production of reactive oxygen species and neuropathology in the brain [47]. Our results demonstrated that genistein or exercise treatment ameliorated the HFHS diet-induced increase in A*β* content. This effect of genistein on the A*β* level has been reported in the AD mouse model and the ob/ob mouse model [37, 48]. However, to the best of our knowledge, this study was the first to show that genistein reduced the accumulation of A*β* using a western-style diet consisting of HFHS to induce obesity and T2DM.

APP is a transmembrane protein found in most tissues. APP is mainly cleaved by *β*- and *γ*-secretase, resulting in the production of A*β* peptides in AD. Under normal conditions, APP is primarily processed by ADAM10, releasing a soluble fragment (sAPP*α*) in a non-amyloidogenic pathway [49]. Activating the non-amyloidogenic pathway by potentiating ADAM10 activity could significantly decrease A*β* release. Also, the product sAPP*α* has neuroprotective properties [49]. Therefore, improving ADAM10 activity represents an attractive approach to prevent the generation of A*β* and favor neurogenesis in AD. We observed a 50% decrease in the levels of ADAM10 protein in HFHS-fed mice compared to control mice. However, treatment with genistein or exercise treatment significantly improved protein levels of ADAM10, which may explain the reduction in A*β* levels observed in the brain of mice fed an HFHS diet. We also observed increased hyperphosphorylated tau and CDK5 levels in HFHS-fed mice. The combination of genistein and exercise training decreased CDK5 levels, which may contribute to the reduction of hyperphosphorylated tau in the mice brain.

BDNF plays an essential role in synaptic plasticity, neuronal growth, and animal behavior. Previous studies have shown that decreased expression of BDNF caused by HFHS feeding was associated with a deficiency in learning performance [50, 51]. The beneficial effects of exercise and an enriched environment on cognitive function are known to be mediated by BDNF [52]. This critical role of BDNF was supported by the observation that blocking the BDNF receptor inhibited the benefits of exercise on hippocampal-dependent learning [53]. In our study, we also observed that protein expression of BDNF increased in exercised-trained mice compared to mice in the HFHS group. Further, increases in BDNF have been reported with genistein in the ovariectomized rat, resulting in improved spatial learning and memory, and decreased seizure activity [54, 55]. However, our results are novel as they provide the first evidence that either genistein and exercise training alone or a combined treatment increased BDNF expression following HFHS feeding in the mouse.

Genistein, because of its similar structure to estrogen, can bind to the ER and previous studies suggest that genistein exerts its effect through estrogenic pathways. For example, genistein enhanced the acetylcholinesterase activity of PC12 cells by binding to the ER [56]. Genistein also exerted anti-apoptotic actions by activating the ER in primary neuronal cells following glutamate exposure [18]. In our study, we found that genistein decreased the expression of caspase-3 induced by an HFHS diet, suggesting decreased apoptotic processes in the brain of mice. Based on these observations, we used the rat pheochromocytoma PC12 cells as an *in vitro* model for neurotoxicity to determine if the ER was involved in this response [57]. To induce neurotoxicity, cells were exposed to a high concentration of glucose and PA, which is known to accumulate in the hypothalamus [58]. Genistein prevented cell death under these conditions and the ER antagonist tamoxifen blocked the protective effects of genistein on cell viability. Moreover, genistein exhibited anti-apoptotic effects expressed as changes in caspase-3 and Bcl2 expression (Figure 7). This is in accordance with previous findings reported by Sonee et al. [59], who indicated that the neuroprotective effect of genistein in cortical cells was attributed to the regulation of the Bcl-2. Our results suggest that the beneficial effects of genistein may occur via the ER in PC12 cells to activate estrogenic neural protective mechanisms. Since there are several subtypes of ER, further studies are warranted to determine if ER-mediated effects of genistein in PC12 cells are specific to a particular ER subtype.

Our data showing greater neuroprotection with combined treatment compared to genistein alone suggests that additional mechanisms other than those mediated via the ER pathway are implicated in this beneficial effect. Indeed, several pathways resulting in different adaptations related to improved brain structure and function are suggested. These adaptations to exercise are generally viewed as structural, hemodynamic, and molecular in nature which occur either through separate or convergent signaling pathways that ultimately reduce disease risk [60–63]. The induction of neurotrophic factors, including BDNF, insulin-like growth factor (IGF), and vascular endothelial growth factor (VEGF), is viewed as a central mechanism associated with the benefits of exercise on brain function [64]. Emerging evidence indicates that exercise training increases BDNF expression via the peroxisome proliferator-activated receptor gamma-1*α*/fibronectin type III domain-containing protein 5 (PGC-1*α*/FNDC5) pathway [65]. Activation of the HSP70/HF*κ*B/IL-6/synapsin 1 axis has been linked to the anti-inflammatory effects of exercise in a rat model of traumatic brain injury [66]. Pathways linked to the benefits of exercise on brain plasticity include the IGF1/PI3K/Akt pathway and the AMPK/SIRT1/PGC-1*α* pathway [67]. Interestingly, increased production of lactate during exercise activates the lactate receptor hydroxycarboxylic acid receptor 1 (HCAR1). Activation of HCAR1 improves the ERK1/2 and Akt signaling, resulting in increased expression of VEGF and angiogenesis in the hippocampus of exercised rats [68].

In summary, chronic HFHS feeding induced changes in the female mouse brain that reflect neurodegenerative states, including AD. Exercise training or genistein treatment offered a modest level of protection against HFHS-induced brain damage. The greatest neuroprotection was provided by combination treatment. This approach was more effective than either treatment alone in decreasing the A*β* load, phosphorylation of tau, and apoptosis.

## Data Availability

The data used to support the findings of this study are included within the article.
